# Measurements of hybrid fertility and a test of mate preference for two house mouse races with massive chromosomal divergence

**DOI:** 10.1186/s12862-018-1322-y

**Published:** 2019-01-16

**Authors:** Sofia A. Grize, Elodie Wilwert, Jeremy B. Searle, Anna K. Lindholm

**Affiliations:** 1 Department of Evolutionary Biology and Environmental Studies, University of Zurich, Winterthurerstrasse 190, 8057 Zurich, Switzerland; 2 Institute for Evolutionary Life Sciences (GELIFES), University of Groningen, Groningen, The Netherlands; 3 Department of Ecology and Evolutionary Biology, Cornell University, Ithaca, NY 14853 USA

**Keywords:** Speciation, Hybridisation, Centric fusion, Hybrid fertility, Hybrid dysfunction, Mate preference, *Mus musculus domesticus*, Robertsonian fusion

## Abstract

**Background:**

Western house mice *Mus musculus domesticus* are among the most important mammalian model species for chromosomal speciation. Hybrids between chromosomal races of *M. m. domesticus* suffer various degrees of fertility reduction between full fertility and complete sterility, depending on the complexity of the chromosomal differences between the races. This complexity presents itself in hybrids as meiotic configurations of chromosome chains and rings, with longer configurations having a stronger impact on fertility. While hybrids with short configurations have been intensively studied, less work has been done on hybrids with very long configurations. In this study, we investigated laboratory-reared wild mice from two chromosomally very different races in Switzerland found in close proximity. Hybrids between these races form a meiotic chain of fifteen chromosomes. We performed a detailed analysis of male and female hybrid fertility, including three generations of female backcrosses to one of the parental races. We also tested for possible divergence of mate preference in females.

**Results:**

While all male F_1_ hybrids were sterile with sperm counts of zero, 48% of female F_1_ hybrids produced offspring. Their litter sizes ranged from one to three which is significantly lower than the litter size of parental race females. When hybrid females were backcrossed to a parental race, half of the offspring resembled the parental race in karyotype and fertility, while the other half resembled the F_1_ hybrids. In the preference test, females of both races indicated a lack of a preference for males of their own karyotype.

**Conclusions:**

Although the fertility of the F_1_ hybrids was extremely low because of the complexity of the chromosomal differences between the races, reproductive isolation was not complete. As we did not find assortative female preferences, we expect that contact between these races would lead to the production of hybrids and that gene flow would occur eventually, as fertility can be restored fully after one backcross generation.

**Electronic supplementary material:**

The online version of this article (10.1186/s12862-018-1322-y) contains supplementary material, which is available to authorized users.

## Background

Speciation is a central topic in evolutionary biology [1], with a need for detailed studies of incipient reproductive isolation of major genetic forms within species through an analysis of hybridisation either in the laboratory or in nature, i.e. where the races make contact at hybrid zones [2]. One characteristic that can define major genetic forms within species is the presence of chromosomal rearrangements; such forms may be termed chromosomal races [3]. Chromosomal rearrangements have the potential to be potent agents promoting reproductive isolation. Mechanical problems during meiosis in hybrids between chromosomal races may lead to subfertility or sterility (hybrid dysfunction) [4, 5]. These include incorrect pairing of parental chromosomes, malsegregation of chromosomes, and errors during crossing-over leading to deletion or duplication of chromosomal regions. In addition, chromosomal rearrangements can also suppress recombination and protect larger parts of the genome from introgression despite hybridisation, facilitating the accumulation of species-specific gene variants involved in Dobzhansky-Muller incompatibilities in these regions [6, 7]. Such incompatibilities may also be related to mate choice.

Chromosomal rearrangements between races and species of mammals include inversions, translocations and fusions/fissions. The western house mouse (*Mus musculus domesticus*) is an excellent model for the study of a very common rearrangement in mammalian karyotype evolution, the Robertsonian (Rb) fusion (= centric fusions): the joining of a pair of acrocentric chromosomes at their centromeres to form a metacentric [8]. While the ancestral karyotype of the western house mouse consists of 2n = 40 acrocentrics, Rb fusions have led to metacentric races—over 100 have been described—with reduced diploid numbers down to 2n = 22 [9, 10]. Hybrids between these races have been shown to suffer a decrease in fitness linked to the complexity of their chromosomal differences [10]. Similar observations have been made for hybrids between chromosomal races in other mammalian species [3, 11], although the house mouse is exceptional for the ease of laboratory studies and availability of genomic and other resources [12].

**Fig. 1:**
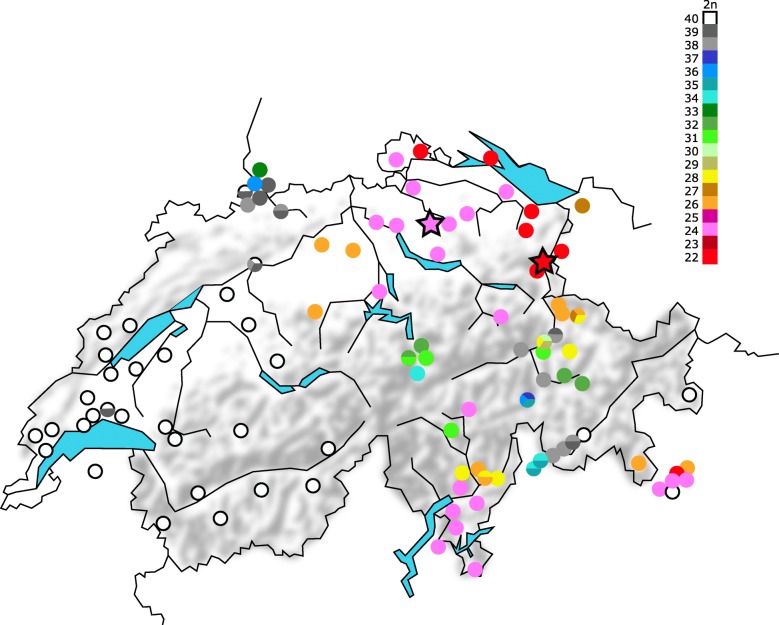
Map of chromosomal variation in house mice in Switzerland and neighbouring areas. The trapping sites of the two races used in this study are marked with stars (pink for CHHN, red for CHBU: see text). The red star represents three closely located trapping sites (within 4 km). Information on the Swiss races was taken from Piálek et al. [9], Hübner [27] and Gropp et al. [64]

The individual metacentric races in house mice occur over very limited geographical areas, although groups of races may occur near to one another, such as in eastern Switzerland (Fig. 1), from where the metacentric races in the present study derive [9]. Two types of heterozygote can be formed on hybridisation of metacentric races in nature or in captivity: simple and complex [3]. Simple heterozygotes are formed when one race has a specific metacentric (e.g. 1.3, formed from chromosomes 1 and 3) and the other one has these chromosomes in the ancestral acrocentric form (1 and 3 in this case). Hybrids are heterozygous for this metacentric, which leads to the formation, during prophase I of meiosis, of a chain of three chromosomes—a trivalent (here 1–1.3–3)—instead of bivalents formed from homologues of either the 1.3 metacentrics or the acrocentrics 1 and 3. Complex heterozygotes are formed when hybridising races have metacentrics that share arms (monobrachial homology, e.g. 1.3 and 3.6). Hybrids in this case produce chains or rings involving four or more chromosomes during meiosis. These configurations are called multivalents (e.g. 1–1.3–3.6–6, a chain of four chromosomes).

Simple heterozygotes with few trivalents (those with one to three have been well studied) show near-normal fertility, while those with a higher number of trivalents (up to nine) are more severely affected and show decreasing fertility with increasing number of trivalents [13, 14]. In contrast, complex heterozygotes usually suffer a more substantial decrease in fertility, with chain meiotic configurations being more detrimental than rings [15, 16]. Although the reduction in fertility is strongly linked to the complexity of the meiotic configurations, there also seems to be an effect of the genetic background [13, 17, 18]. Also, female heterozygotes are generally less severely affected than males [10, 15]. The most detailed studies of fertility of hybrids between chromosomal races, with substantial quantitative data, relate to simple heterozygotes and complex heterozygotes for short chain configurations. There are data for both males and females, but substantially more on males [13, 14, 17, 19–24].

**Fig. 2:**
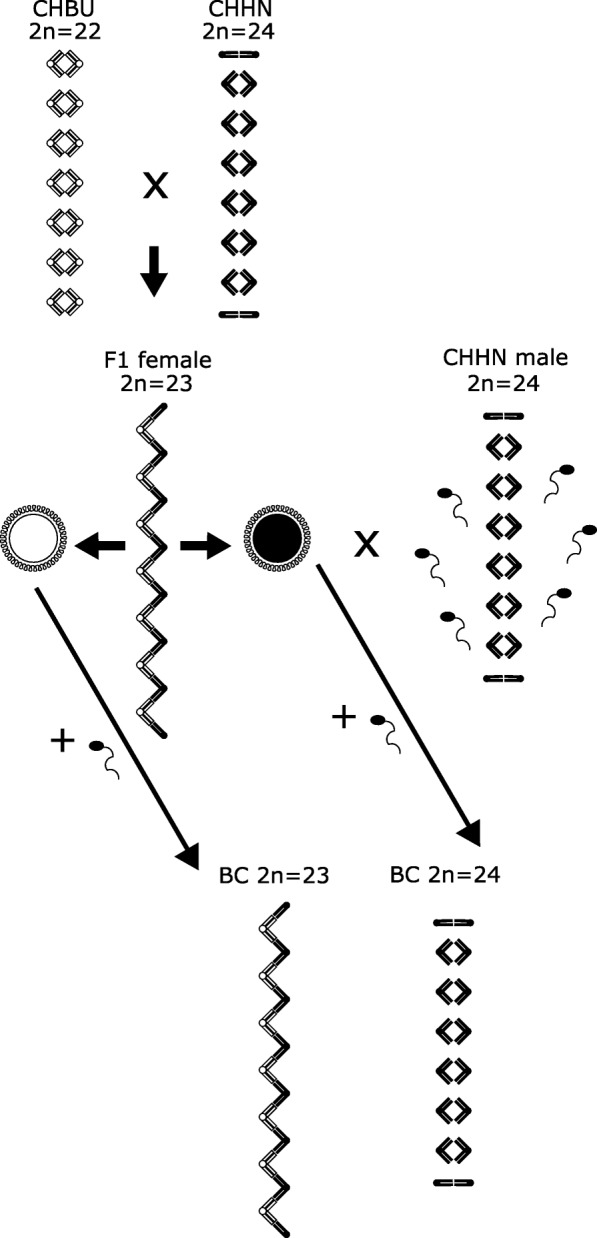
Expected inheritance of a meiotic chain of fifteen chromosomes considering parental, F_1_ and backcross individuals. F_1_ female mice are expected to produce gametes containing either chain chromosomes from their CHBU parent (white), or chain chromosomes from their CHHN parent (black). The fusion of these oocytes with sperm from CHHN males (black) should lead to backcross offspring (BC) with the hybrid karyotype (2n = 23) or the CHHN karyotype (2n = 24). Chromosomes not involved in the chain need not segregate according to race of origin

Here we carry out detailed fertility studies on both male and female complex heterozygous F_1_ hybrids between two chromosomal races that produce a very long chain of fifteen chromosomes at meiosis. We test the degree of F_1_ hybrid sterility by performing sperm counts on males and backcrossing females. We repeated this procedure for the offspring of the backcrosses. As F_1_ hybrids are expected to produce only two types of balanced gametes (Fig. 2), F_1_ females should have offspring with two karyotypes: half should match the F_1_ karyotype, the other half that of the parental race used in the backcross.

In addition to the effect of chromosomal differences, geographic separation itself could lead to divergence of behaviour, which could act as a premating barrier if the races were to come into contact. Such premating divergence has been described between the western house mouse and its sister subspecies, the eastern house mouse *Mus musculus musculus* (e.g. [25, 26]). Therefore, we tested for female preference when given a choice between males of the two races.

## Methods

### Mice

**Fig. 3:**
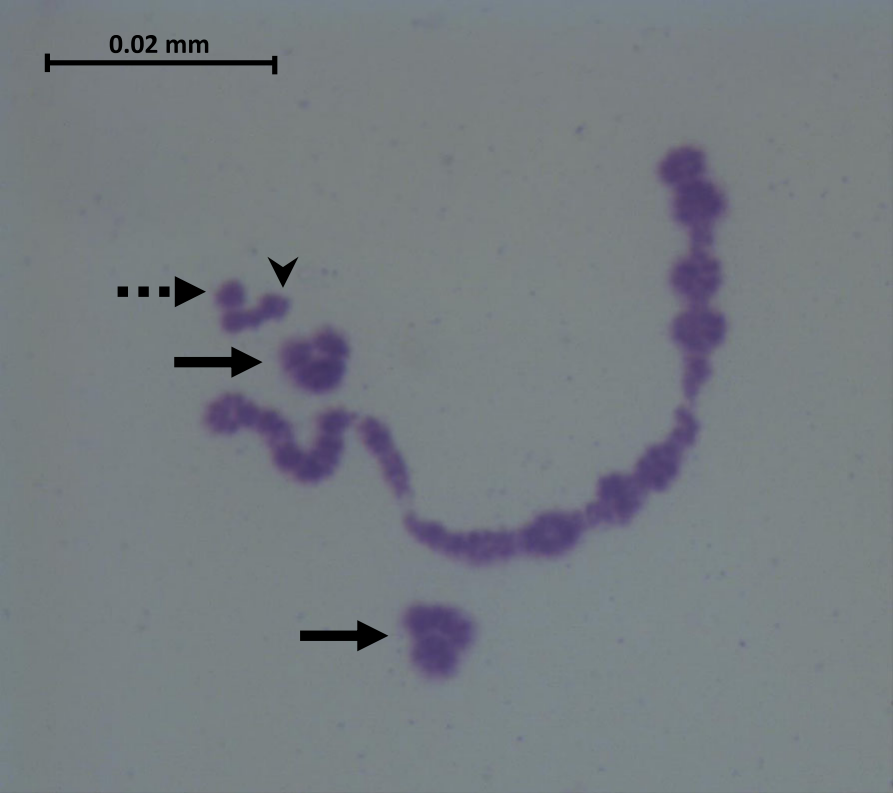
Diakinesis preparation from testes of a male F_1_ hybrid between the races CHHN and CHBU. The chain of fifteen chromosomes is visible in the centre, with two sets of metacentric bivalents above and below (filled arrows), the chromosome 19 bivalent (dashed arrow) and the sex bivalent (arrowhead)

Mice from two very different and previously little studied chromosomal races from Switzerland were used in this study (Fig. 1). Following the chromosome and race nomenclature in Piálek et al. [9], they are the Buchs race CHBU with 2n = 22 (1.18 2.5 3.6 4.12 7.15 8.16 9.14 10.17 11.13 [27]), and the Hünikon race CHHN with 2n = 24 (1.3 2.8 4.12 5.7 6.15 9.14 10.11 13.16 [27]). F_1_ hybrids between these races are expected to have the following configurations during meiosis I: a multivalent chain of fifteen chromosomes (18–18.1–1.3–3.6–6.15–15.7–7.5–5.2–2.8–8.16–16.13–13.11–11.10–10.17–17) and three autosomal bivalents formed from homologues of 4.12, 9.14 and 19, respectively, as well as the sex-bivalent (Fig. 3). All animals used in this study were laboratory-reared and are descended from wild mice (CHHN and CHBU: four and five generations in the laboratory, respectively). CHHN mice were trapped in Illnau, Switzerland (see König & Lindholm [28] for a description of the study population), while CHBU mice were trapped in farms and stables in the nearby locations of Grabs, Buchs and Haag, Switzerland, between 2010 and 2014 (trapping locations shown in Fig. 1). The approximate direct distance between Illnau and Buchs is 60 km. Previous sampling in this region found CHHN and CHBU races at sites as close as 30 km apart, but with no sampling in between [27].

Mice were kept either singly in Makrolon Type II cages or in groups in Makrolon Type III cages (Indulab). Groups consisted of same-sex siblings or of a breeding pair. Cages contained bedding, cardboard housing and paper towels as nesting material. Food and water was available ad libitum. Animals in the fertility experiment were kept under a light:dark cycle of 14:10 with lights on at 05.30 CET. Animals in the mate preference test were kept under a reversed cycle with lights on at 17.30 CET.

### Crosses

**Fig. 4:**
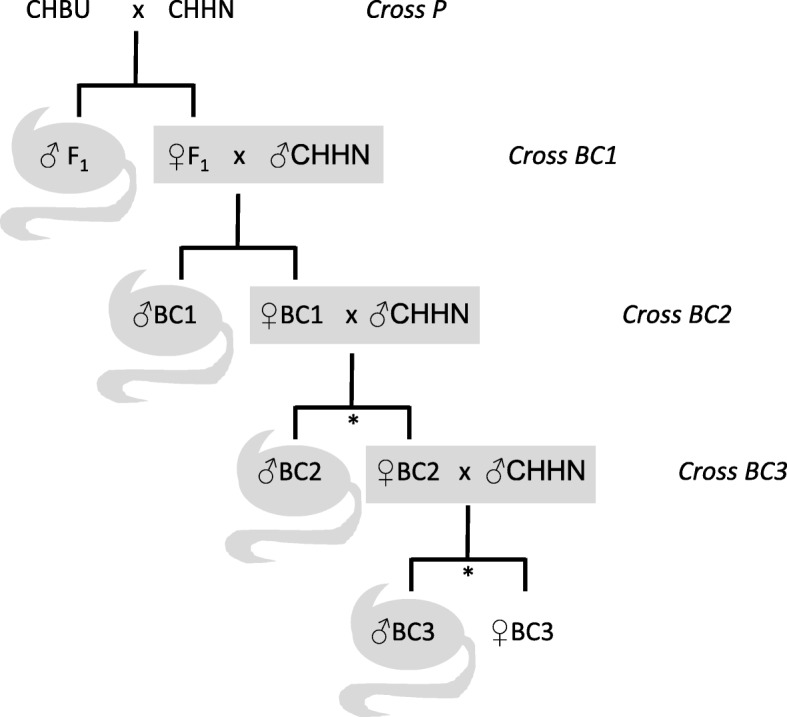
Crossing scheme applied between mice that derive from the Buchs (CHBU) and Hünikon (CHHN) races. Male offspring were sacrificed and sperm counts were performed. Female offspring were backcrossed to a male of the CHHN race. After the first backcross, not all offspring were processed equally (stars). Fewer offspring were analysed if their mother was fully fertile (see text)

The first generation (P) consisted of 14 crosses between mice from CHBU and CHHN (five ♀CHBUx♂CHHN, nine ♀CHHNx♂CHBU). One to nine litters per breeding pair were obtained. We assessed the fertility of hybrid offspring. Where available, two male offspring per breeding pair were used for sperm counts. On average two female offspring (2.2 ± 0.7; mean ± SD) per breeding pair were backcrossed to unrelated CHHN males. This process was repeated for the following generations, with males sacrificed for sperm counts and females backcrossed to CHHN males (Fig. 4, Table 1). Females in backcrosses were kept with males for up to five months (12–159 d) in order to also assess litter size of females with very low fecundity. However, if breeding pairs showed high levels of aggression within those five months, they were separated prematurely but not excluded from the analysis. Thirty-two (74%) backcrossed females were separated prematurely (after 76 ± 37 d; details in Additional file 1). In addition, if females had two litters with litter sizes of at least five pups each, we considered them as fully fertile and terminated the cross. We performed sperm counts on two male offspring from such females but did not backcross any of their female offspring. Mice were checked for pups on a weekly basis.

**Table 1:** Details of animal numbers in all experimental crosses (see Fig. 4)

			Number of offspring born				Number of offspring weaned			Expected		Verified	
Cross	Number of crosses	Duration (mean ± SD) [days]	Male	Female	Sex unknown (died as pups)	Total	Male	Female	Total	2*n* = 23	2*n* = 24	2*n* = 23	2*n* = 24
P	14	137.7 ± 63.0	162	163	35	360	162	163	325	325	0	7	0
BC1	31	84.1 ± 43.2	8	15	0	23	4	12	16	8	8	4	11
BC2	10	62.8 ± 33.9	54	54	3	111	54	54	108	?	?	3	3
BC3	2	81.0 ± 52.3	9	4	0	13	9	4	13	?	?	1	0

Diploid chromosome number was verified for a sample of offspring (see text)

### Karyotype analysis

Mitotic chromosome spreads were prepared according to Ford [29] and stained with Giemsa. For each individual, at least twenty clear and complete metaphases were counted. According to our expectations outlined in the Introduction (Fig. 2), all F_1_ mice were expected to have 2n = 23 chromosomes. We verified this by counting chromosomes of seven randomly chosen F_1_. After backcrossing to a male of the parental CHHN race with 2n = 24, we expected offspring of either 2n = 23 (hybrid karyotype) or 2n = 24 (parental CHHN karyotype). Thus, we made chromosome counts for offspring of all female 2n = 23 hybrid mice (this included offspring from BC1, BC2 and BC3 crosses). Chromosomes from offspring from 2n = 24 hybrid females (this included offspring from BC2 and BC3 crosses) were not counted.

Offspring were classified into three categories: (1) F_1_ hybrids with 2n = 23, (2) BC.23 hybrids with 2n = 23, resulting from backcrosses to CHHN, and (3) BC.24 hybrids with 2n = 24 also resulting from backcrosses to CHHN.

### Fertility of males

Male offspring were sacrificed at an age of nine weeks (mean ± SD, 65 ± 3 d). Twenty-five male F_1_ hybrids were analysed. As controls, 12 male CHBU and 17 male CHHN of similar age were used (62 ± 3 d, 64 ± 5 d, respectively). The right cauda epididymis was processed as described in Hauffe & Searle [22]. Spermatozoa were counted in both chambers of a Neubauer Improved haemocytometer. Sperm estimates were calculated by dividing the number of sperm by the volume of the haemocytometer squares counted (ten squares of 0.04 mm^2^ with 0.1 mm depth or, if no sperm was visible in that area, the whole 9 mm^2^) and then by multiplying with the total solution used to dilute the epididymis (2 ml). The results from both chambers were averaged for each male. The resulting value estimates the total number of sperm in one cauda epididymis. Other data collected from males were as follows: body mass after euthanasia, epididymis mass (mean of separate measurements of right and left complete epididymides), testis mass (mean of separate measurements of right and left testes), mass of seminal vesicle and coagulating gland (as these organs could not be reliably separated, they were weighed together; half the mass of combined left and right dissected as one entity) and preputial gland mass (half the mass of combined left and right dissected as one entity). Male offspring of backcrosses were processed in the same fashion (*N* = 21) and included male offspring from fully fertile BC2 and BC3 females (two per female). To increase sample size for these comparisons, we set up additional crosses in exactly the same way as the P crosses to provide 35 additional males (mean age 60 ± 3 d; 17 F_1_, 12 CHBU, 6 CHHN).

We applied linear mixed-effects models (LMM) with parent pair as a random effect in order to take into account that siblings were included in the analysis. Independent variables were category of male (CHBU, CHHN, F_1_, BC.23, BC.24) and body mass. We used the lme4, lsmeans and lmerTest packages in R 3.3.0 [30–33]. Denominator degrees of freedom were approximated using the Satterthwaite method. We tested the assumption of normality for all models in this study with a Normal Q-Q plot of the residuals. However, the assumption of normality was not well satisfied for sperm number. Thus we first applied a Fisher’s exact test to analyse the difference in prevalence of males with and without sperm depending on whether a meiotic chain of fifteen was expected or not (F_1_ and BC.23 vs CHBU, CHHN and BC.24). Then we applied the LMM described above on the male categories with sperm (CHBU, CHHN and BC.24) after performing a common logarithmic transformation on sperm number.

In order to demonstrate the presence of a meiotic chain configuration of fifteen chromosomes in F_1_ hybrids, we made diakinesis preparations from the testes of two male F_1_ hybrids according to Bulatova et al. [34].

### Fertility of females

Female fertility was estimated by whether or not pups were born, and by the number of offspring in the female’s first litter (31 F_1_, 12 BC). For a subset of these females (19 F_1_, 12 BC), the number of implantation scars in the uterus was counted post mortem from fresh tissue [35, 36]. In order to attribute any decrease in fertility to the females rather than to the males, we performed sperm counts on the latter. As estimates for number of offspring in females of the parental races, we used data from the same breeding system of our laboratory that had furnished P mice and CHHN backcross males for this experiment. From all first litters born to females from within-race breeding pairs over the period 2013–2016, we randomly selected 12 litters from CHBUxCHBU crosses, and 12 litters from CHHNxCHHN crosses. We used a one-way ANOVA to compare litter sizes between experimental females and controls. Additionally, we performed a LMM on the data of the experimental females with litter size as the dependent variable, category of female and body mass as independent variables, and parent pair as a random effect to correct for the presence of full-siblings. The same R packages were used as for the analysis of male fertility. Plots were created using ggplot2 [37].

### Female preference test

**Fig. 5:**
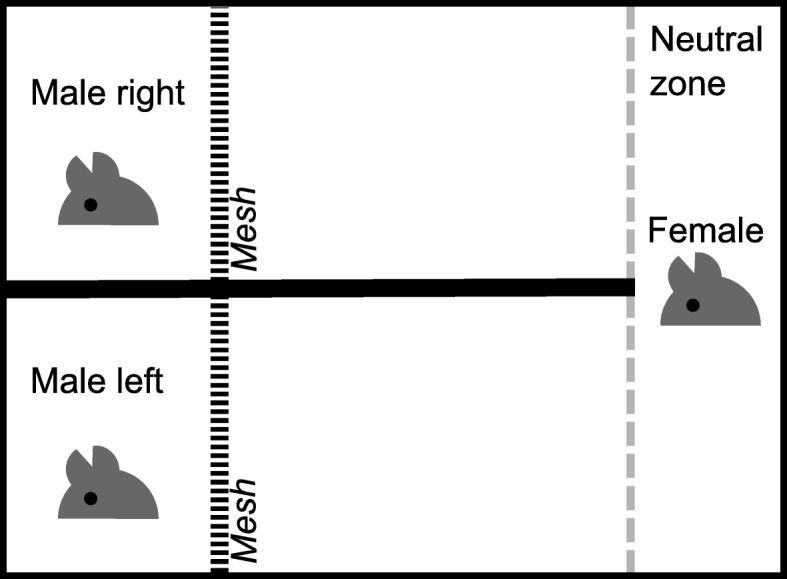
Diagram of the preference test apparatus. The thick dashed black line indicates the mesh grid. The dashed grey line demarks the neutral zone and does not represent a physical barrier

Female mate preference was tested in an apparatus of 75x55cm containing three chambers: two small ones for the males (each 25.5 × 19.5 cm) and a large one for the female (Fig. 5) [38–40]. The male chambers were separated by an opaque wall which extended into the female’s chamber. Male and female chambers were separated by a mesh (0.5 cm^2^ grids) that allowed vocal, visual and tactile interactions between the male and the female but prevented copulation. Males were unable to see or access each other. Fifty-eight mature but sexually inexperienced female mice (two to four months old) from the races CHBU (*N* = 30) and CHHN (*N* = 28) were tested for mate preference using pairs of CHBU and CHHN males, also sexually inexperienced. Males were allocated randomly to the left and right male chambers. Twenty-two of thirty-six (61%) male pairs were used twice, once with a CHBU female and once with a CHHN female. All experiments were performed between 11.00 and 15.00 CET in a room with reversed light cycle (light:dark cycle of 14:10, lights on at 17.30 CET; habituation period of two weeks) and filmed from above under red light in the dark without the presence of an observer. Males were first placed into their respective chambers during a habituation period of 15 min. Afterwards, a female was placed into the neutral zone (see Fig. 5) and allowed to roam freely within her chamber. We only used females at the pre-oestrus and oestrus stage of their cycle, verified by vaginal smears as described in Byers et al. [41]. After the experiment, the movements of female and male mice were scored blindly with respect to their race using the event recorder BORIS [42]. Measurements were started after the female had exited the neutral zone on both male sides once [43, 44], and lasted 30 min [39, 40]. We measured the time females spent with their nose within 1 cm of the mesh [39, 40]. In addition, we measured the time males spent with their nose within 1 cm of the mesh.

For the statistical analysis, the proportion of time P a female spent near the mesh of the CHHN male was calculated as (time at mesh of CHHN male)/(time at mesh of CHHN male + time at mesh of CHBU male). LMMs were performed following logit transformation of the response variable time proportion P. Transformations were performed using the boot package in R [45, 46]. Fixed effects were female race, difference in male body mass, difference in male age, and difference in time males spent near the mesh. All differences between males were calculated as (CHHN value) − (CHBU value). These differences were centred, and scaled to standardise variation. Random effects were ID of the male pair used and the chamber side the CHHN male was placed in (right or left, to correct for a potential preference of the female for a specific side).

## Results

### Karyotype analysis

The seven analysed F_1_ hybrids had a diploid chromosome number of 2n = 23 with 17 metacentrics and 6 acrocentrics. Offspring from F_1_ females had either 2n = 23 with 17 metacentrics and 6 acrocentrics (*N* = 4), or 2n = 24 with 16 metacentrics and 8 acrocentrics (*N* = 11). This was also the case for offspring from females with 2n = 23 from the BC1 and BC2 generation (offspring with 2n = 23: N = 4, offspring with 2n = 24: *N* = 3, Table 1).

### Fertility of males

**Fig. 6:**
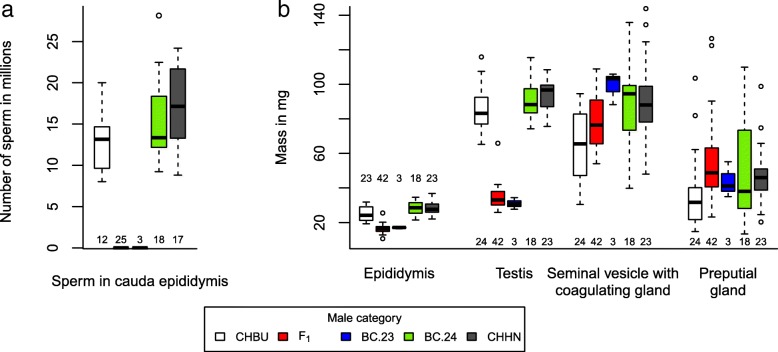
Boxplot representation of measurements taken from male mice for the comparison of fertility. **a** Number of sperm in the cauda epididymis and (**b**) the mass of epididymis, testis, seminal vesicle with coagulating gland, and preputial gland were compared between males of the CHBU race, the CHHN race, F_1_ hybrids between CHBU and CHHN, and offspring resulting from backcrosses of hybrids to CHHN (see Fig. 4). Offspring from backcrosses were grouped according to their diploid chromosome number (BC.23 for 2n = 23, BC.24 for 2n = 24). The boxplot boundaries indicate the minimum, the first quantile, the median, the third quantile and the maximum. Outliers are shown as circles. The sample sizes are listed below or above the corresponding boxplots. Note that in the male fertility analysis, the category BC.24 includes offspring from fully fertile BC2 and BC3 females (two per female)

All F_1_ and BC.23 males had sperm counts of zero, which was significantly different from the males of the remaining categories (Fisher’s exact test, *p* < 10^− 15^; Table 2; Fig. 6a). For the males of these remaining categories, which all had sperm counts well above zero, the linear mixed effects model did not indicate a significant effect of male category but did indicate a significant effect of body mass (Tables 3 and 4). In the remaining models containing all five male categories, category of male was highly significant for testis mass and epididymis mass (Table 2; Fig. 6b). Category of male was marginally significant for the mass of the seminal vesicle with coagulating gland but not for the preputial gland mass (Table 3). Testis mass and epididymis mass were significantly lower for F_1_ and BC.23 males (Table 4). However, the mass of the seminal vesicle with coagulating gland and the preputial gland mass were not significantly lower for F_1_ or BC.23 males (Table 4). There was a significant positive relationship between body mass and testis mass, epididymis mass, mass of the seminal vesicle with coagulating gland, and preputial gland mass (Table 3). The relationship between body mass and the response variables was examined in detail using linear regression models, which indicated that for sperm number, testis mass and epididymis mass, the positive relationship was limited to sperm producing males (CHHN, CHBU, BC.24; Additional file 2).

**Table 2:** Mean and standard deviation for male fertility measurements for all groups

	CHHN	CHBU	F_1_	BC.23	BC.24
Body mass in g	25.0 ± 1.9	22.1 ± 3.0	23.7 ± 3.0	26.3 ± 1.3	23.6 ± 2.4
Number of sperm in cauda epididymis (10^3^)	17,019 ± 5074	12,935 ± 3594	0 ± 0	0 ± 0	15,247 ± 5018
Testis mass in mg	93.22 ± 9.75	84.77 ± 12.11	34.56 ± 6.59	30.95 ± 3.33	90.91 ± 10.79
Epididymis mass in mg	28.24 ± 3.93	25.04 ± 4.40	16.27 ± 2.25	17.02 ± 0.61	28.43 ± 3.99
Mass of seminal vesicle with coagulating gland in mg	91.23 ± 22.87	65.60 ± 19.46	78.64 ± 15.65	99.10 ± 9.49	88.28 ± 25.19
Preputial gland mass in mg	47.39 ± 16.64	36.12 ± 21.45	54.22 ± 23.11	43.8 ± 10.35	48.84 ± 28.64

**Table 3:** Results of ANOVA analyses of effect of male category and body mass on male fertility

Response variable	Explanatory variable	df numerator	df denominator	F	*p*-value
Cauda sperm	Male category	2	25.19	1.12	0.34
	Body mass	1	39.04	4.47	0.04
Testis mass	Male category	4	74.75	210.29	< 10^−15^
	Body mass	1	103.29	15.81	< 0.001
Epididymis mass	Male category	4	72.16	63.10	< 10^−15^
	Body mass	1	103.00	23.80	< 10^−5^
Seminal vesicle and coagulating gland	Male category	4	73.13	2.63	0.04
	Body mass	1	103.69	45.79	< 10^−9^
Preputial gland	Male category	4	72.13	2.01	0.10
	Body mass	1	93.78	22.93	< 10^−5^

Parent pair was used as a random effect to correct for the inclusion of siblings in the dataset. For all response variables except cauda sperm, male categories included the CHBU parental race, the CHHN parental race, F_1_ between CHBU and CHHN, offspring from backcrosses to CHHN with 2n = 23, and offspring from backcrosses to CHHN with 2n = 24. In the model for cauda sperm, only male categories with sperm were tested (CHBU, CHHN, and offspring from backcrosses to CHHN with 2n = 24, see text)

**Table 4:** Pairwise comparisons of reproductive parameters for different categories of males

Response variable		CHBU-CHHN	F_1_-CHHN	BC.23-CHHN	BC.24-CHHN	BC.23-F_1_
Number of sperm in cauda epididymis (10^3^)	Estimate	−2549.9	na^*^	na^*^	−456.8	na^*^
	SE	1666.1	na^*^	na^*^	1626.5	na^*^
	df	26.6	na^*^	na^*^	27.4	na^*^
	t	−1.42	na^*^	na^*^	−0.27	na^*^
	*p*-value	0.29	na^*^	na^*^	0.94	na^*^
Testis mass in mg	Estimate	−4.89	−57.09	−63.64	0.26	−6.55
	SE	3.03	2.60	5.36	3.16	5.26
	df	71.4	67.1	100.7	68.3	101.0
	t	−1.61	−21.92	−11.87	0.08	−1.24
	*p*-value	0.32	< 10^−4^	< 10^−4^	1.00	0.53
Epididymis mass in mg	Estimate	−1.49	−11.21	−11.21	0.98	0.00
	SE	1.09	0.94	1.88	1.13	1.85
	df	68.8	62.8	99.5	64.6	99.9
	t	−1.36	−11.97	−5.96	0.86	0.00
	*p*-value	0.46	< 10^−4^	< 10^−4^	0.77	1.00
Mass of seminal vesicle with coagulating gland in mg	Estimate	−14.11	−7.01	4.92	2.11	11.93
	SE	5.64	4.85	9.88	5.88	9.70
	df	69.4	64.9	100.4	66.4	100.8
	t	−2.50	−1.45	0.50	0.36	1.23
	*p*-value	0.05	0.41	0.93	0.97	0.54
Preputial gland mass in mg	Estimate	−1.07	10.99	−8.52	6.00	−19.51
	SE	6.50	5.52	12.57	6.69	12.35
	df	70.5	66.8	101.9	65.7	101.8
	t	−0.16	1.99	−0.68	0.90	−1.58
	*p*-value	1.00	0.16	0.86	0.75	0.33

Pairwise comparisons of least square means of male categories using Dunnett’s method for *p* value adjustment were applied to the LMMs with male category and male body mass as explanatory variables, and the ID of the males’ parents as a random effect. Estimate and standard error of sperm number were transformed back before insertion into the table. *For number of sperm, only fertile male categories were included in the model (see text)

### Fertility of females

**Fig. 7:**
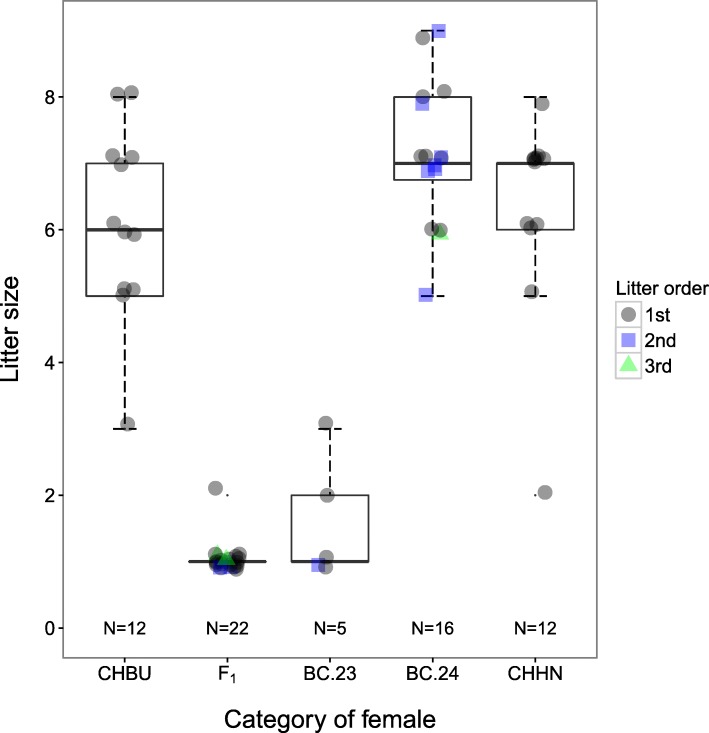
Number of offspring in each litter for females that reproduced. For the experimental crosses, all litters born are shown. For the controls, only the first litter is shown. The boxplots are overlain with the individual data points

Female hybrids were backcrossed to males of the CHHN chromosomal population over three generations (crosses BC1–BC3, Fig. 4, animal numbers in Table 1). They gave birth to their first litters between 3 and 20 weeks after pairing (mean ± SD, 8.0 ± 5.0 weeks). From the 31 F_1_ females which were all backcrossed to CHHN males, 15 (48%) of them produced offspring (Table 1). From the ten BC1 females and the two BC2 females backcrossed, all produced offspring. In the LMM for size of first litter, female category was significant (F_4, 39.7_ = 63.61, *p* < 10^-15^). F_1_ and BC.23 females both had significantly smaller first litters than females of the categories CHHN, CHBU and BC.24 but did not differ significantly from one another (CHHN: 6.3 ± 1.5, CHBU: 6.1 ± 1.4, F_1_: 1.1 ± 0.3, BC.23: 1.8 ± 1.0, BC.24: 7.5 ± 0.9; Table 5, Fig. 7).

**Table 5:** Pairwise comparisons of size of first litter for different categories of females using Dunnett’s method

Contrast	Estimate of difference in litter size between contrasted races	SE	df	t	*p*-value
CHBU–CHHN	−0.38	0.51	37.06	−0.74	0.83
F_1_–CHHN	−5.20	0.48	37.08	−10.80	< 10^−4^
BC.23–CHHN	−4.50	0.62	45.94	−7.27	< 10^−4^
BC.24–CHHN	1.33	0.53	42.63	2.49	0.06
BC.23–F_1_	0.70	0.62	46.00	1.13	0.61

The LMMs applied contained female category (see text) as the only explanatory variable and the identity of the females’ parents as a random effect to control for including siblings in the data

**Fig. 8:**
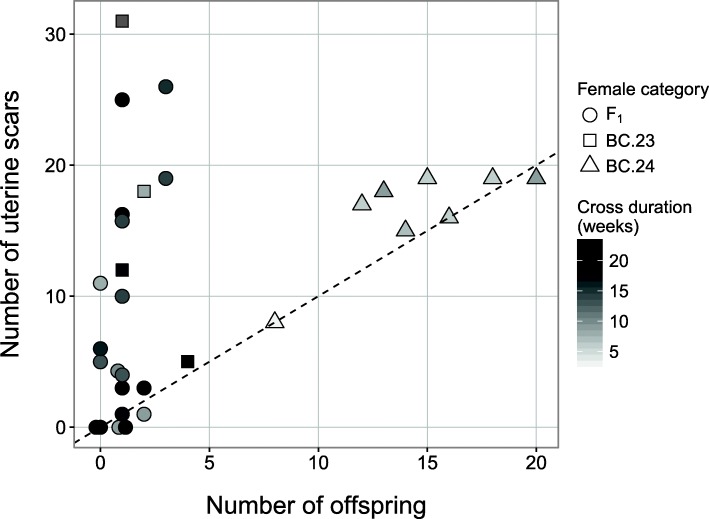
Total offspring number versus number of uterine scars for the experimental females (F_1_’s and backcrosses). Females are separated into three categories: F_1_ between the races CHBU and CHHN (*N* = 19), females from backcrosses with 2n = 23 (BC.23, *N* = 4), and females from backcrosses with 2n = 24 (BC.24, *N* = 8). The dashed line indicates where the number of scars equals the number of total offspring i.e. every embryo that was implanted survived until birth. Females above the dashed line had more scars than offspring, indicating the loss of implanted embryos. The comparison of cross duration for the different females shows that it was not a major explanatory factor for differences in scar and offspring number

In the additional comparisons between experimental females, BC.24 females reproduced sooner than F_1_ and BC.23 females (mean ± SD time to first litter: BC.24: 3.5 ± 0.8 weeks, F_1_: 8.0 ± 5.0 weeks, BC.23: 8.5 ± 2.1 weeks; Wilcoxon-Mann-Whitney test, BC.24–F_1_: W = 99, *p* = 0.012; BC.24–BC.23: W = 32, *p* = 0.008). We also examined 31 females for the presence of scars in the uterus as an indication of successful embryo implantation. Generally, we expect fertile females to have the same number of scars as offspring if all implanted embryos survived. In contrast, an excess of scars would indicate that not all of the implanted embryos survived. Twenty-two (71%) of the females examined had more scars than offspring (Fig. 8). While the number of scars in BC.24 females was very similar to the number of total offspring, the number of scars in many F_1_ and BC.23 females greatly exceeded the number of offspring, with the number of scars ranging from zero to thirty-one (Fig. 8). Three females without offspring had multiple scars. Four females had one scar less than offspring number, suggesting that scar numbers may have been underestimated.

The CHHN males used in the backcrosses had a mean ± SD number of sperm in the cauda epididymis of 23.1 ± 12.1 million, which is in the range of the sperm counts for CHHN control males in the male hybrid fertility analysis (Fig. 6).

### Female preference test

**Fig. 9:**
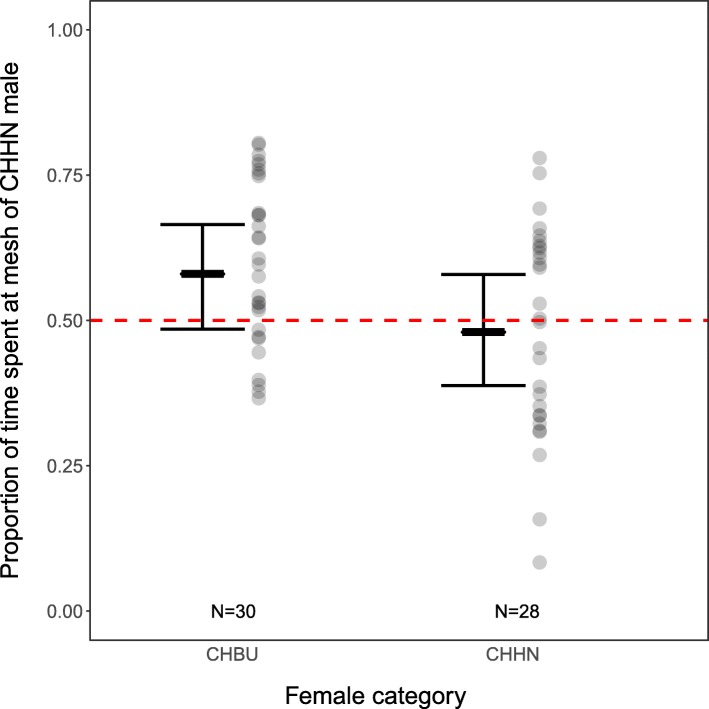
Relative time that females spent near the CHHN male when presented with males of both races. A value of 0.5 (dashed line) represents equal amount of time spent near either male. Positive values indicate more time spent near the CHHN male, negative values more time spent near the CHBU male. The grey circles represent the actual data points, while the bars represent the linear mixed model’s estimate of the means (thick horizontal bars) and the 95%-confidence intervals for females of either race

Neither CHHN nor CHBU female mice spent significantly more than 50% of their time at the mesh with males of any race (CHHN females: *t* = − 0.35, *p* = 0.77; CHBU females: *t* = 1.65, *p* = 0.30). However, CHBU females spent a greater proportion of their time close to the CHHN male than did CHHN females (Table 6, Fig. 9). Difference of male age influenced time near the CHHN male: the older the CHHN male was relative to the CHBU male, the less time females spent near him. There was no effect of time males spent near the mesh, nor of differences in body mass (Table 6). Male age did not correlate with body mass (linear regression, F_1, 114_ = 0.30, *p* = 0.58). On average, females spent 34.5 ± 11.9% of the experimental time near the meshes.

**Table 6:** Linear mixed model of logit transformed relative time proportion spent by female near CHHN male

		β estimate	SE	df	t	*p*-value
Female race	CHHN (intercept)	–0.07	0.2	1.52	–0.35	0.77
	CHBU	0.38	0.19	52.38	2.01	0.05
Δ Male body mass		0	0.1	52.56	0	1
Δ Male age		–0.24	0.1	52.64	–2.44	0.02
Δ Male time spent at mesh		0.18	0.09	52.44	1.87	0.07

## Discussion

Studying hybridisation between major genetic forms within species is one approach to better understand the process of diversification. In this study, we investigated the effect of chromosomal rearrangements, specifically Robertsonian fusions, on the fertility of hybrids between two such genetic forms—chromosomal races—of house mice. F_1_ hybrids (2n = 23) between the races CHBU (2n = 22) and CHHN (2n = 24), which show substantial chromosomal differences, form a multivalent chain of fifteen chromosomes during meiosis. We report in a detailed analysis that male F_1_ hybrids are sterile while female F_1_’s have low fertility. Backcrossing of female F_1_ to males of the CHHN race resulted in the restoration of the CHHN karyotype and full fertility in half of the offspring. However, we consider hybrid fertility overall to remain relatively low as the occurrence of fertile offspring would be strongly limited by the very low F_1_ fertility, and the probability of a sterile backcross offspring. In the preference test, females did not show a preference for males of either race.

### Hybrid fertility

Spermatogenesis was disrupted in the male F_1_ hybrids, as they had sperm counts of zero with a lower mass of the testis and epididymis but normally sized seminal vesicle (measured with coagulating gland) and preputial gland. Low sperm counts linked to low testes mass have been previously described, e.g. in Flachs et al. [47]. This tight relationship between sperm count and mass of the testis and epididymis is expected for physiological reasons, as sperm is located in these organs and should thus influence their mass.

Unlike the males, F_1_ female hybrids were partially fertile. Half of the F_1_ females produced litters upon backcrossing. However, it took them longer than for parental race females until their first litter was born and the number of offspring was significantly decreased. Most hybrid females had litter sizes of only one offspring instead of the usual six to seven observed in mice of the parental races. F_1_ females also had an excess of uterine scars that indicated that they had lost multiple foetuses. The cause is most likely aneuploidy. Aneuploid embryos and foetuses have been previously found in pregnant mice heterozygous for metacentrics [48]. In addition, extremely high rates of nondisjunction, ranging up to 100%, have been described in female complex heterozygotes [15, 22]. Nondisjunction results in monosomic and trisomic zygotes after fertilisation. While monosomics die before implantation, trisomics usually survive past implantation and leave visible scars in the uterus [49, 50]. Thus, the scars in the F_1_ females could indicate the loss of trisomic foetuses.

The fertility results of these F_1_ hybrids concur with previous studies on complex heterozygotes with long chain configurations during meiosis [15, 16, 18, 51, 52]. In general, male-limited sterility with disrupted spermatogenesis has been observed. However, these studies are mostly qualitative, some without detailed information on sample sizes or the races that were crossed. In addition, the fertility of females was not always investigated. We aimed to contribute a detailed and complete study of complex heterozygote fertility. The most similar study to ours is that of Capanna et al. [15], in which fertility of F_1_ hybrids between the Poschiavo race (2n = 26) and the Cittaducale race (2n = 22) were studied. These complex heterozygotes possess a chain of seventeen and a chain of three chromosomes during meiosis. Preparation of spermatocytes from nine male hybrids and histological inspection of the testes of eight males indicated that the formation of mature sperm was absent. In contrast, 13 oocyte preparations of meiosis II could be collected from eight female hybrids. Of the ten female hybrids that were crossed to fertile males during four to five months, only two litters with one offspring each were obtained. Both of these offspring were sterile, although no information on karyotype was given. These results are similar to our findings, although female F_1_’s between the CHBU und CHHN races appear to have a higher fertility. This could be due to them having only one, slightly shorter, meiotic chain with fifteen chromosomes or to a more similar genetic background, as these races are geographically more closely located than the Poschiavo and Cittaducale races.

Thus in Capanna et al. [15] and the current study, gametogenesis in complex heterozygotes with very long chain configurations was affected differently in males and females. This accords with Haldane’s Rule [53], and matches findings in mice that mutations affecting meiotic chromosome alignment and segregation generally have stronger effects in sperm than in oocytes, with cessation of spermatogenesis and continued oogenesis [54]. Garagna et al. [19] studied oogenesis directly and found that in heterozygous metacentric mice, egg follicles that successfully pass the first stages of oogenesis also finish their development. In addition, they described a reduction in follicle number for complex heterozygote females with long meiotic chains, indicating a shorter reproductive lifespan. We thus expect CHBUxCHHN F_1_ females not only to suffer from reduced litters and an increased number of aneuploid offspring due to nondisjunction, but to also suffer a decrease in reproductive lifespan due to a smaller supply of follicles.

We expected that backcrossing female F_1_ (2n = 23) to CHHN males (2n = 24) would lead to two types of offspring (Fig. 2): those with a hybrid karyotype (2n = 23) and those with the CHHN karyotype (2n = 24). This pattern was corroborated in our findings. In addition, the fertility analyses showed that when the CHHN karyotype was restored in the offspring, their fertility was normal and equal to that of mice of the parental races. In contrast, offspring with the F_1_ hybrid karyotype displayed the fertility of the F_1_ hybrids with sterility in males and low fertility in females. Under the assumption of no recombination (a very simplified scenario), we would expect backcross offspring with 2n = 24 chromosomes to not only regain the parental karyotype, but also the genetic material from the chain chromosomes of that race, while offspring with 2n = 23 would be genetically heterozygous for all chain chromosomes (Fig. 2). However, there is strong support that recombination is obligate in mice and other mammals [55] and studies on simple heterozygotes between mouse chromosomal races have shown that a minimum of one recombination event is maintained [56–58]. While complex heterozygotes with long chains have not yet been studied, it would seem probable that recombination rates remain close to the seemingly obligate one recombination event per chromosome arm. Thus, under the assumption of recombination, offspring from backcrosses should be genetic hybrids, even if they have the karyotype of a parental race. As no intermediate fertility values were observed, karyotype and fertility restoration seem to be directly related, suggesting that the decrease in fertility in hybrids between CHBU and CHHN is entirely due to chromosomal heterozygosity instead of genic incompatibilities or other genic effects. Even closely related individuals such as full siblings showed opposite fertility patterns if their karyotypes differed. These results differ from findings of studies in which the fertility of hybrids between mouse chromosomal races varied and indicated a possible involvement of a genic effect [13, 17, 22]. Nevertheless, as chromosomal races in the house mouse all belong to the same subspecies, the accumulation of Dobzhansky-Muller incompatibilities seems unlikely.

### Female preference

We did not find a preference for males of the same chromosomal race in our behavioural test, in which differences in the duration of proximity of the female to each male was used as a proxy for mate choice, a common experimental design [59], and in which differences in male behaviour, age and body mass were accounted for in the analysis. These results suggest that females would not discriminate between males of compatible versus incompatible karyotype in their mate choice. In a system of chromosomal races on Madeira, assortative preferences were also absent, although the races did show a divergence of their preference [60]. Furthermore, behavioural differences between neighbouring metacentric races have been previously described [61–63]. In our study, we found a trend for an effect of male behaviour and a significant effect of age on the females’ behaviour. Thus, females may have been responding to other cues, e.g. of male quality or interest. In addition, we cannot exclude that laboratory rearing had an effect on the expression of female preferences or male traits.

As there does not seem to be assortative mating preferences between the CHBU and CHHN races, we expect contact to lead to the formation of F_1_ hybrids and, as female F_1_’s are not sterile, to allow gene flow between the races, potentially opposing the evolution of assortative mating. As fertility is further restored in part of the backcross offspring, we expect gene flow to be higher than expected when only considering F_1_ fertility.

## Conclusions

This study presents a detailed report on fertility of laboratory-reared hybrids between two chromosomal races of house mice with a high complexity of chromosomal differences. Despite the long meiotic multivalent of fifteen chromosomes in F_1_ hybrids and sterility of male F_1_’s, female F_1_’s remained fertile, although to a low degree. Backcrosses of these females to males of one of the parental races resulted for part of the offspring in restoration of the karyotype to that of the parental race and full fertility, indicating that the decrease in fertility of hybrids is likely entirely due to karyotype. The remaining offspring had the karyotype and low fertility of F_1_ hybrids, with males being sterile and females having low fertility. A preference test on females of the parental races indicated a lack of preference for chromosomally compatible males. Thus, these races are expected to hybridise in the event of contact. While the chromosomal differences have the potential to promote reproductive isolation between the races through the extremely low fertility of F_1_ hybrids, the marginal fertility of female F_1_’s combined with the restoration of fertility in part of the offspring from backcrosses could lead to more gene flow than expected based solely on the length of the chain configuration in hybrids.

## Additional files

Additional file 1: Duration of female backcrosses. (PDF 11 kb)

Additional file 2: Linear regressions between male body mass and the variables sperm number, testis mass, epididymis mass, mass of seminal vesicle with coagulating gland and mass of preputial gland. (PDF 136 kb)

Additional file 3: Data of male fertility. (XLSX 20 kb)

Additional file 4: Data of female fertility. (XLSX 13 kb)

Additional file 5: Data of litter sizes for all litters of hybrid and control females. (XLSX 14 kb)

Additional file 6: Data of sperm counts of males used in female backcrosses. (XLSX 10 kb)

Additional file 7: Data of female preference test. (XLSX 17 kb)
