# Supplementary material for: Measurements of hybrid fertility and a test of mate preference for two house mouse races with massive chromosomal divergence
Source: BMC Evol Biol. 2019 Jan 16;19:25. doi: 10.1186/s12862-018-1322-y (PMC6335807; doi:10.1186/s12862-018-1322-y)
Supplement: Supplementary file 1 — Duration of female backcrosses. (PDF 11 kb) [file 12862_2018_1322_MOESM1_ESM.pdf]

Additional file 1: Duration of female backcrosses

Females in backcrosses (categories F_1, BC.23, BC.24) were mated with males for up to five months. However, 74% of those females were separated prematurely because of high levels of aggression between them and their mates. In addition, females which were considered fully fertile (after having two litters each with at least five pups) were also separated prematurely (all were category BC.24). The fertility differences between these categories were not associated with differences in cross length (Supplementary Fig. S1).

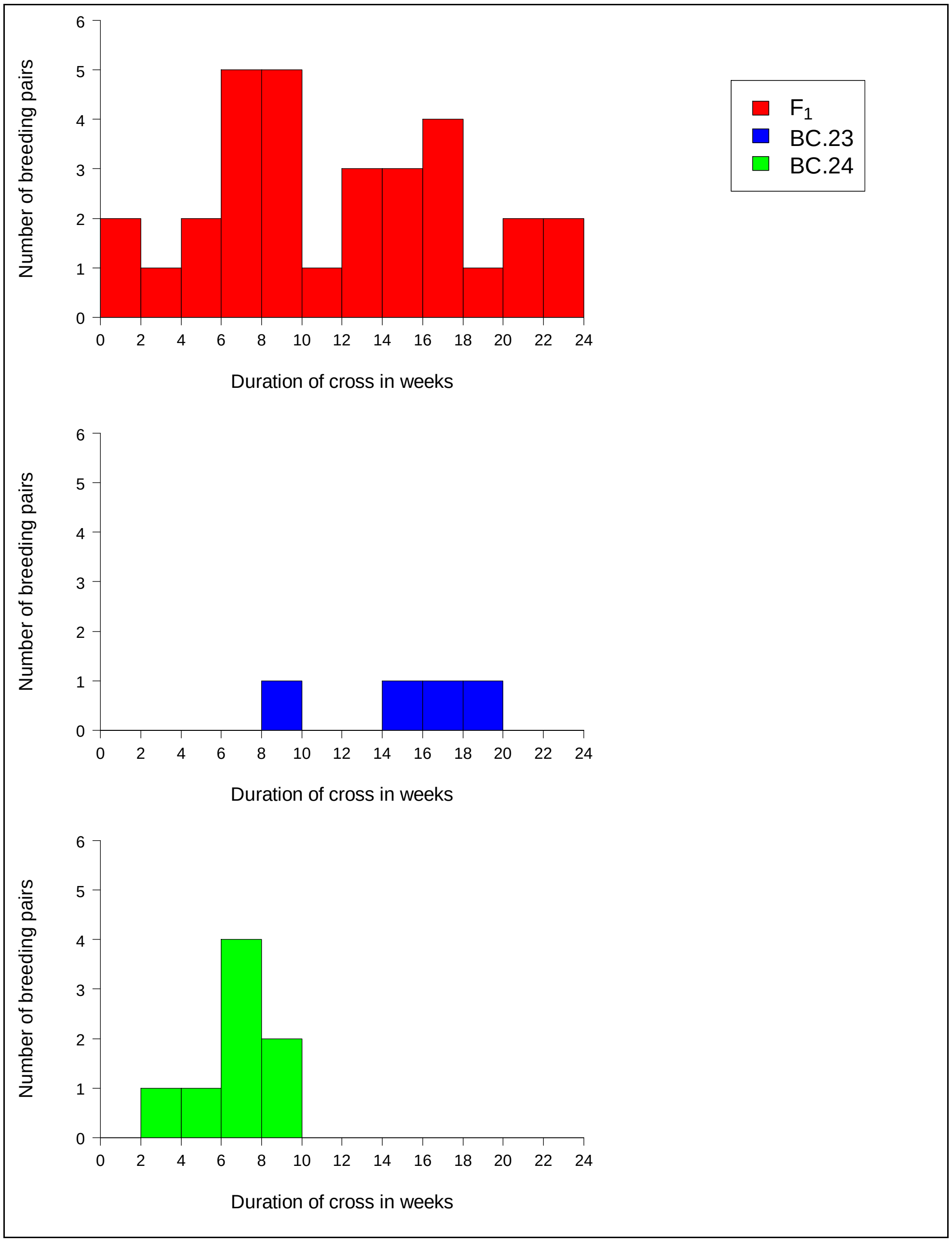

Supplementary Fig. S1 Histogram of the cross duration for females that were backcrossed to CHHN males, separated by category (bin width: 2 weeks).
