# Supplementary material for: Measurements of hybrid fertility and a test of mate preference for two house mouse races with massive chromosomal divergence
Source: BMC Evol Biol. 2019 Jan 16;19:25. doi: 10.1186/s12862-018-1322-y (PMC6335807; doi:10.1186/s12862-018-1322-y)
Supplement: Supplementary file 2 — Linear regressions between male body mass and the variables sperm number, testis mass, epididymis mass, mass of seminal vesicle with coagulating gland and mass of preputial gland. (PDF 136 kb) [file 12862_2018_1322_MOESM2_ESM.pdf]

Additional file 2: Linear regressions between male body mass and the variables sperm number, testis mass, epididymis mass, mass of seminal vesicle with coagulating gland and mass of preputial gland

We performed linear regressions between body mass and number of sperm, testis mass, epididymis mass, mass of seminal vesicle with coagulating gland, and mass of preputial gland, all measured as described in the Methods. Males were grouped as hybrid karyotype (F_1 and BC.23) and parental karyotype (CHBU, CHHN, BC.24). The relationships were significant for parental karyotype in all cases (sperm: $F_{1,45}=6.4$, $p<0.02$; testis: $F_{1,63}=26.1$, $p<10^{-5}$; epididymis: $F_{1,62}=48.9$, $p<10^{-8}$; seminal vesicle with coagulating gland: $F_{1,63}=57.5$, $p<10^{-9}$; preputial gland: $F_{1,63}=15.4$, $p<10^{-3}$). However, the relationships were only significant for the hybrid karyotype in the regressions for mass of seminal vesicle with coagulating gland ($F_{1,43}=22.5$, $p<10^{-4}$) and mass of preputial gland ($F_{1,43}=9.6$, $p<0.01$). Plots are shown in Supplementary Fig. S2.

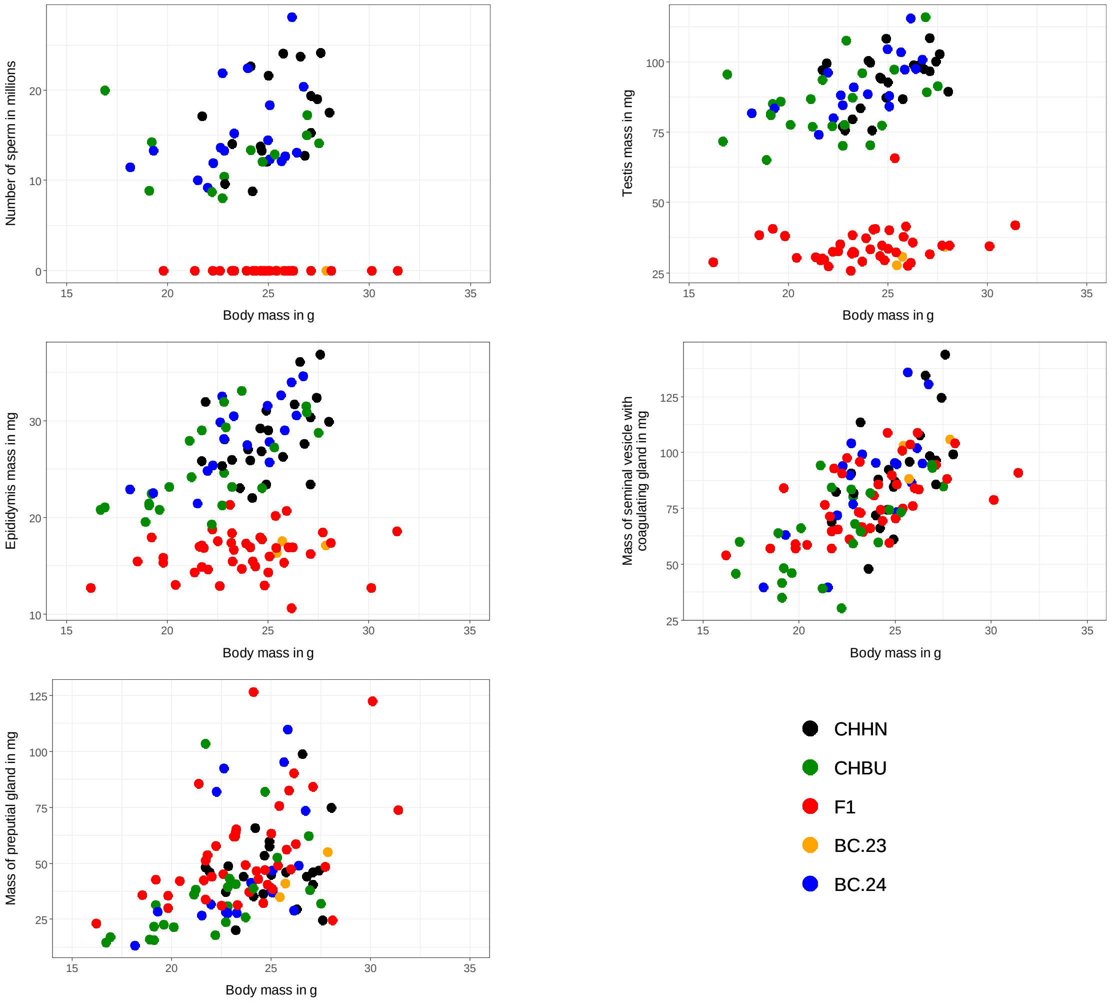

Supplementary Fig. S2 Relationship between males’ body mass and sperm number and mass of male reproductive organs.
